# Ultra Deep Sequencing of *Listeria monocytogenes* sRNA Transcriptome Revealed New Antisense RNAs

**DOI:** 10.1371/journal.pone.0083979

**Published:** 2014-02-03

**Authors:** Sebastian Behrens, Stefanie Widder, Gopala Krishna Mannala, Xiaoxing Qing, Ramakanth Madhugiri, Nathalie Kefer, Mobarak Abu Mraheil, Thomas Rattei, Torsten Hain

**Affiliations:** 1 Department für Computational Systems Biology, Universität Wien, Wien, Austria; 2 Institute für Medizinische Virologie, Justus-Liebig Universität Giessen, Giessen, Germany; 3 febit biomed GmbH, Heidelberg, Germany; 4 Life Technologies GmbH, Darmstadt, Germany; 5 Institute für Medizinische Mikrobiologie, Justus-Liebig Universität Giessen, Giessen, Germany; Charité-University Medicine Berlin, Germany

## Abstract

*Listeria monocytogenes*, a gram-positive pathogen, and causative agent of listeriosis, has become a widely used model organism for intracellular infections. Recent studies have identified small non-coding RNAs (sRNAs) as important factors for regulating gene expression and pathogenicity of *L. monocytogenes*. Increased speed and reduced costs of high throughput sequencing (HTS) techniques have made RNA sequencing (RNA-Seq) the state-of-the-art method to study bacterial transcriptomes. We created a large transcriptome dataset of *L. monocytogenes* containing a total of 21 million reads, using the SOLiD sequencing technology. The dataset contained cDNA sequences generated from *L. monocytogenes* RNA collected under intracellular and extracellular condition and additionally was size fractioned into three different size ranges from <40 nt, 40–150 nt and >150 nt. We report here, the identification of nine new sRNAs candidates of *L. monocytogenes* and a reevaluation of known sRNAs of *L. monocytogenes* EGD-e. Automatic comparison to known sRNAs revealed a high recovery rate of 55%, which was increased to 90% by manual revision of the data. Moreover, thorough classification of known sRNAs shed further light on their possible biological functions. Interestingly among the newly identified sRNA candidates are antisense RNAs (asRNAs) associated to the housekeeping genes *purA*, *fumC* and *pgi* and potentially their regulation, emphasizing the significance of sRNAs for metabolic adaptation in *L. monocytogenes*.

## Introduction

*Listeria monocytogenes* is a Gram-positive, facultative intracellular pathogen, which is responsible for a foodborne infection, listeriosis, a rare but serious disease. It has become the prime model organism for intracellular pathogens [1]. Small non coding RNAs (sRNAs) have been proposed to play an important role in the pathogenicity of *L. monocytogenes* and some lead to attenuated infections when disabled [2], [3]. These studies also showed that antisense transcription is common in *L. monocytogenes* [2], [3]. Beside short antisense RNAs (asRNAs), a new type of long antisense RNAs (lasRNAs) functioning as an mRNA as well as antisense RNA that regulate adjacent genes at the level of transcription, was proposed [4].

Over the last decade reduced costs for high throughput sequencing (HTS) technologies facilitate the thorough and unbiased research of bacterial transcriptomes at an ever increasing rate [5]–[7]. As a result, identification of small non coding RNAs in all bacterial species have been reported [8]–[11]. Large numbers of small non coding RNAs have been found in both Gram-negative [12], [13] and Gram-positive [14], [15] bacteria. In particular *L. monocytogenes* has been subject to an extensive number of transcriptome studies using macro-/microarrays, Illumina GAIIx or Roche GS FLX sequencing platforms [2]–[4], [16]–[20]. The SOLiD sequencing platform used in this study, provides a very high throughput sequencing method with increased base calling accuracy due to its unique ‘color coded’ di-base sequencing technique [21].

Here we report the thorough reevaluation of the small RNA transcriptome of *L. monocytogenes* with increased coverage. A large HTS transcriptome dataset containing transcriptomic data of *L. monocytogenes* grown under intracellular and extracellular conditions was the basis of this study. The transcriptomic data was generated using the SOLiD HTS platform and consists of a total of 21 million reads. In this study a newly developed computational pipeline was used to identify and classify sRNAs. Furthermore, this computational pipeline leads to the discovery of nine yet unknown small non coding RNA candidates of *L. monocytogenes*.

## Materials and Methods

### Bacterial and cell culture and RNA extraction

The strain of *L. monocytogenes* EGD-e [22] and the murine P388D1 macrophages were used for cell infection and RNA extraction as reported recently for this study [2]. The strain *L. monocytogenes* EGD-e used in this study was grown in brain heart infusion (BHI) broth (VWR) overnight at 37°C with shaking at 180 rpm (Unitron, Infors). Overnight cultures were diluted 1∶50 in 20 ml fresh BHI broth using a 100 ml Erlenmeyer flask and were incubated at the same conditions mentioned above until mid-exponential phase (OD_600 nm_ 1.0). Bacteria were added to P388D1 murine macrophage cells monolayer at a multiplicity of infection (MOI) of ten bacteria per eukaryotic cell.

For RNA extraction from extracellularly grown *L. monocytogenes*, we used aliquots of 0.5 ml from the same bacterial culture used to infect P388D1 macrophages. The bacterial cells were treated with 1.0 ml RNA protect (Qiagen) for 5 min and were collected by centrifugation for 10 min (8000×g) and subsequently stored at −80°C until use. RNA extraction from intracellularly grown *L. monocytogenes* in macrophages, 4 h post infection, was performed as described previously [33] [23]. Briefly, infected host cells were lysed using cold mix of 0.1% (wt/vol) sodium dodecyl sulfate, 1.0% (vol/vol) acidic phenol and 19% (vol/vol) ethanol in water. The bacterial pellets were collected by centrifugation for 3 min (16000×g).

Total RNA was extracted using miRNeasy kit (Qiagen) with some modifications. The collected pellets were washed with SET buffer [50 mM NaCl, 5 mM EDTA and 30 mM Tris-HCl (pH 7.0)]. After centrifugation at 16000×g for 3 min pellets were resuspended in 0.1 ml Tris-HCl (pH 6.5) containing 50 mg/ml lysozyme (Sigma), 25 U of mutanolysin (Sigma), 40 U of SUPERase (Ambion), 0.2 mg of proteinase K (Ambion) and incubated at 37°C for 30 min at 350 rpm. QIAzol (Qiagen) was added, mixed gently and incubated for 3 min at room temperature. An additional incubation at room temperature was done after adding 0.2 volume chloroform followed by centrifugation at 16000×g at 4°C for 15 min. The aqueous phase, containing RNA, was transferred to a new collection tube and 1.5 volumes of 100% ethanol was added and mixed thoroughly. The probes containing RNA were transferred into columns supplied with the miRNeasy Kit (Qiagen) and treated according to the manual including an on-column DNase digestion (RNase-Free DNase, Qiagen). RNA was eluted by RNase-free water and stored at −80°C until needed. The quantity of the isolated total RNA was determined by absorbance at 260 nm and 280 nm, and the quality was assessed using Nano-chips for Agilent's 2100 Bioanalyzer. For detection and estimation of the small RNA fraction within the isolated total RNA, a small RNA-chip (Agilent) was used, which visualizes RNAs with sizes ranging from 20 to 150 nucleotides.

### RNA sequencing

6 µg of total RNA of the intracellular and the extracellular sample was used as starting material. The quality was checked by Nanodrop and Agilent Pico RNA Chip. Both samples were prepared in parallel for all three different size ranges from <40 nt, 40–150 nt and >150 nt.

>150 nt size fractionation library preparation. 2.5 µg of total RNA of the sample was rRNA depleted using the Ribo Minus Bacteria Module (Invitrogen Corporation) and purified with the RiboMinus Concentration Module (Invitrogen Corporation) with a modified protocol to keep all RNA transcripts <200 nt. After the rRNA depletion the samples were checked on the Pico RNA Chip from Agilent showing remaining rRNA in the sample. However, due to the small starting amount the rRNA depletion couldn't be repeated. Subsequently, the RNA was treated with Tobaco-Acid-Pyrophosphatase (TAP) from epicenter ® for 1.5 h at 37°C and purified with the RiboMinus Concentration Module. Fragmentation of the RNA was done with RNaseIII (LifeTechnologies, RNA-Seq Kit) (37°C, 10 min) and again purified with the RiboMinus Concentration Module. The samples were dried with a Speed Vacuum Pump, resuspended in 3 µl of nuclease-free water and the SOLiD Adapters were ligated (65°C, 10 min; 16°C, 5 min). After ligation, mRNAs were reversely transcribed into cDNA with Array Script ™ Reverse Transcriptase (Life Technologies, RNA-Seq Kit) and purification was done with Qiagen's MinElute PCR Purification Kit, eluting in 20 µl nuclease-free water. cDNA fragments between 150 nt and 250 nt (fragmented transcripts + adaptor sequences) were isolated from a 6% TBE Urea Gel (Novex-System, Invitrogen). cDNA from gel slices was amplified with 16 PCR cycles using the same 5′-Primer for each sample and two different 3′-Primers including the barcode sequences (SOLiD Multiplexing Barcoding Kit 01-16). Purification was done with the Micro PCR Purification Kit (Invitrogen Corporation).

<40 nt and 40–150 nt size fractionation library preparation. 3.5 µg of total RNA of the sample was enriched with the flashPAGE Fractionator (Ambion) with a modified protocol (runtime 40 min) in order to enrich RNA molecules up to 150 nt. Purification was done with the flashPAGE Clean up Kit (Ambion). The samples were dried with a Speed Vacuum Pump, resuspended in 3 µl of nuclease-free water and the SOLiD Adapters were ligated (65°C, 10 min; 16°C, 5 min). After ligation, small RNAs were reverse transcribed into cDNA with Array Script™ Reverse Transcriptase, (Life Technologies, RNA-Seq Kit) and purification was done with Qiagen's MinElute PCR Purification Kit, eluting in 20 µl. Afterwards, the small RNAs (cDNA) were size-selected on a 10% TBE Urea Gel (Novex-System, Invitrogen). Different size ranges were collected from the gel (60–80 nt, 80–120 nt, 120–150 nt) and amplified with 16 PCR cycles using the same 5′-Primer for each sample and four different 3′-Primers including the barcode sequences (SOLiD Multiplexing Barcoding Kit 01-16). PCR purification was done with the Micro PCR Purification Kit (Invitrogen Corporation). A total of six purified and barcoded DNA libraries were analyzed on a HS-DNA Chip on the Agilent Bioanalyzer 2100 and subsequently pooled in equimolar amounts.

Next generation sequencing. The pooled libraries were diluted to a concentration of 60 pg/µl. DNA was amplified monoclonally on magnetic beads in an emulsion PCR. Emulsions were broken with butanol and the remaining oil was washed off the double-stranded beads. DNA molecules on the bead surface were denatured to allow hybridization to polystyrene enrichment beads. Using a glycerol cushion null beads can be separated from the templated beads. In an additional denaturation step, the templated beads were separated from the enrichment beads. The 3′-ends of the DNA molecules on the bead's surface were enzymatically modified for deposition on the sequencing slide. The beads were loaded onto a slide and sequenced on a SOLiD 3 Plus analyzer producing reads of 50 nt length.

### Data processing

**Figure 1:**
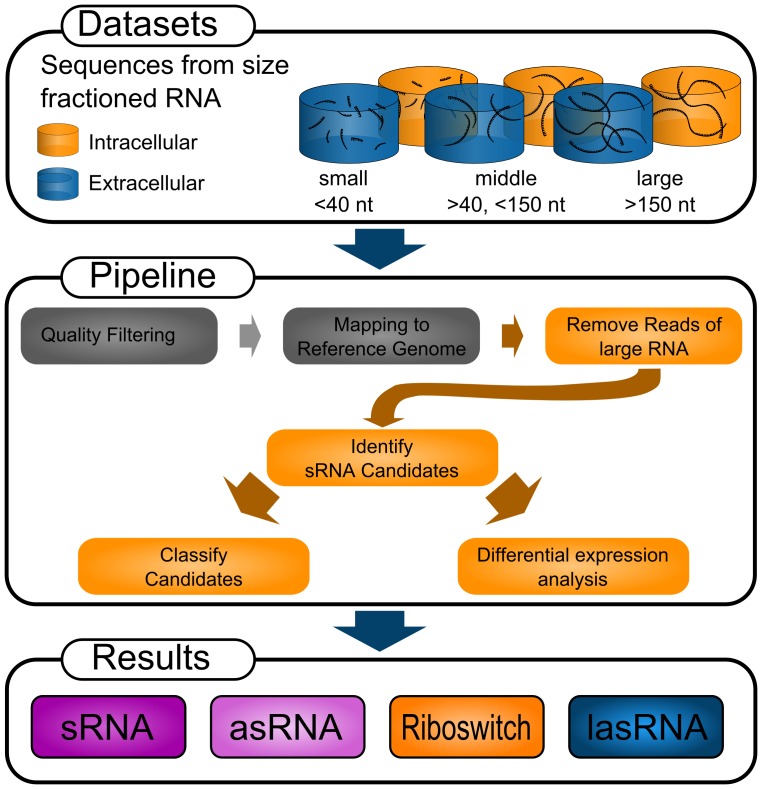
Schematic representation of the main computational pipeline used in this study and its input and output. The pipeline is optimized to work with sequence data from fractionated RNA samples containing RNA fragments of different lengths. Data gathered under various conditions can also be used for differential expression analysis. For this study we used data from the SOLiD High Throughput Sequencing (HTS) platform, but the pipeline will also process data from all major HTS platforms. The individual steps within the pipeline are colored either gray or orange representing steps for which existing software was used and newly implemented features respectively. The result of the pipeline will be lists of pre-classified sRNA candidates.

To identify and characterize new candidates as well as to compare known sRNAs to our transcriptome data set we implemented a novel computational pipeline. See Fig. 1 for an overview of all processing steps. We made use of the specific data set properties including the SOLiD sequencing technique, producing short and “color coded” sequencing data and data, split into two growth conditions and three RNA size fractions. The two growth conditions representing extracellular and intracellular lifestyle of *L. monocytogenes* and the size fractions containing extracted RNA of different lengths, namely <40 nt, 40–150 nt and >150 nt. The fragmentation will allow for a fine-grained differentiation between degradation products of large RNA molecules and independently expresses sRNAs.

Fig. 1 gives an overview over this pipeline, for a detailed description of the pipeline and the used parameters see supplementary file S1. In brief, the pipeline first maps reads onto a reference genome using a short read mapper. We compared different mapping programs for this purpose, including SHRiMP, Bfast and BWA, and performed a parameter evaluation to achieve an optimal mapping. Based on this evaluation we chose BWA as mapper with a maximum mismatch rate per read of 2. Our pipeline then utilizes annotation data as well as coverage information from different size fractions to filter the dataset and identify large RNA molecules expressed on the genome. The *L. monocytogenes* genome annotation was obtained on 28/09/2011 from NCBI RefSeq: (ftp://ftp.ncbi.nih.gov/genomes/Bacteria/Listeria_monocytogenes_EGD_e_uid61583/). Our pipeline considers reads of smaller fractions that were aligned to a region in which a larger fraction indicated a transcript as degradation products originating from the larger transcript. After masking of all known transcripts as well as degradation products, an expanding window algorithm identified putative novel sRNA candidates within the remaining transcriptome.

The pipeline also implements a number of downstream analysis tools. These include an automatic comparison tool to identify equivalent sRNAs between different size fractions, samples, or studies, enabling us to quickly compare other studies of the same organism or differential expression between experimental conditions. An automated classification system is also part of the pipeline to classify transcription start sites, asRNAs, and classical sRNAs. A last tool enables a more fine-grained statistical analysis of differential expression between two given datasets. It visualizes the data in an MA-plot and lets the user select custom thresholds depending on average expression, to fine-tune the significance of the differential expression.

The pipeline as well as the corresponding java program ncFinder are accessible at http://fileshare.csb.univie.ac.at/ncFinder_associated_files/pipeline.tgz and http://fileshare.csb.univie.ac.at/ncFinder_associated_files/ncFinder.zip respectively.

### Differential expression analysis

We used NOIseq [37] to perform a differential expression analysis. The method based on the assumption, that on average, the expression is similar between case and control. We used RPKM to normalize the data and required a p-value of <0.1 for a locus to be considered differentially expressed. We summarized the results in supplemental table S2.

### Conservation analysis

Mauve was used to check the conservation status of the nine sRNAs. Multiple genome alignments were calculated using default parameters for the following *Listeria* species: *Listeria monocytogenes* serovar 1/2a EGD-e (NC_003210), *Listeria innocua* CLIP11262 (NC_003212.1), *Listeria welshimeri* serovar 6b str. SLCC5334 (NC_008555.1), *Listeria seeligeri* serovar 1/2b str. SLCC3954 (NC_013891.1), *Listeria ivanovii* subsp. *ivanovii* PAM 55 (NC_016011.1) and *Listeria marthii* FSL S4-120 (NZ_CM001047.1).

### Oligonucleotides

Oligonucleotides that were used for northern blot hybridization and qRT-PCR are listed in supplementary table S3.

### Northern blot analysis

RNA samples (∼30 µg were normalized to 5S rRNA hybridization signals) were denatured for five minutes at 65°C in loading buffer containing 50% deionized formamide, separated on urea-polyacrylamide (10%) gels, and transferred to nylon membrane by electroblotting in a semi dry blotter according to the manufacturer's recommendations. Membranes were hybridized with gene-specific [γ-^32^P]-end-labeled oligodeoxy-ribonucleotides [24].

### 5′end labeling of primers with [γ-^32^P]ATP

DNA probes were generated by 5′-end-labelling of RNA –specific oligonucleotides with [γ-^32^P] ATP which is described elsewhere [24]. All probes were purified on G25 Microspin columns (GE healthcare) and probes were further used for hybridization.

### Quantitative real-time PCR analysis

Total RNA was isolated from the *L. monocytogenes* EGD-e grown in BHI medium and macrophages as described above. RNA isolation was followed by production of strand-specific cDNA from 1 µg total RNA and SuperScript II Reverse Transcriptase (Invitrogen) by using primers designated _a (see supplementary table S3) which is complementary to the asRNA or the *lmo2673*. The generated cDNA probes were subjected to quantitative real-time PCR in a final volume of 25 µl using primers designated _b (see supplementary table S3) and QuantiTect SYBR Green PCR kit (Qiagen) according to the manufacturer's instruction. A standard curve was generated for the used primer pairs using different copy numbers of genomic DNA from EGD-e (see supplementary table S3). For each primer pair a negative control (water), RNA sample without reverse transcriptase (to determine genomic DNA contamination) and a sample with known amount of copy numbers (to test the efficiency of the reaction) were included as controls during cDNA quantification. All samples after real-time PCR were run on a 1.5% agarose gel to verify that only a single band was produced.

### Statistical data analysis

All infection experiments for qRT-PCR and northern blots analysis were performed in a minimum of three biological experiments. Significant differences between two values were compared with a paired Student's t-test. Values were considered significantly different when the *p* value was less than 0.05 (*p*<0.05).

### Accession number

RNA sequencing data have been deposited to EBI (http://www.ebi.ac.uk/), accession number PRJEB4644.

## Results

To investigate the transcriptome of *L. monocytogenes* RNA was extracted from bacteria grown either in BHI (extracellular growth) or in murine macrophages (intracellular growth). The RNA was then fractionated into 3 fractions with cutoffs <40 nt, 40–150 nt and >150 nt respectively to aid unambiguous differentiation between sRNA and degradation products of larger RNA molecules. Subsequently RNA extracts were sequenced using SOLiD sequencing technology. A total of 21 million reads over six sequencing runs were obtained. Reads from the fraction containing RNA <40 nt were trimmed to 30 nt length since we expected a high false sequencing error at the 3′ end of these reads. We applied quality filtering to the reads to ensure that reads which very likely contain sequencing errors are not used in further analysis. A total of 71% of reads were retained after filtering. Detailed filtering counts are listed in supplementary table S4. Application of our sRNA pipeline on the data yields a total of 711 sRNA candidates for further analysis.

### Transcription start site detection

**Figure 2:**
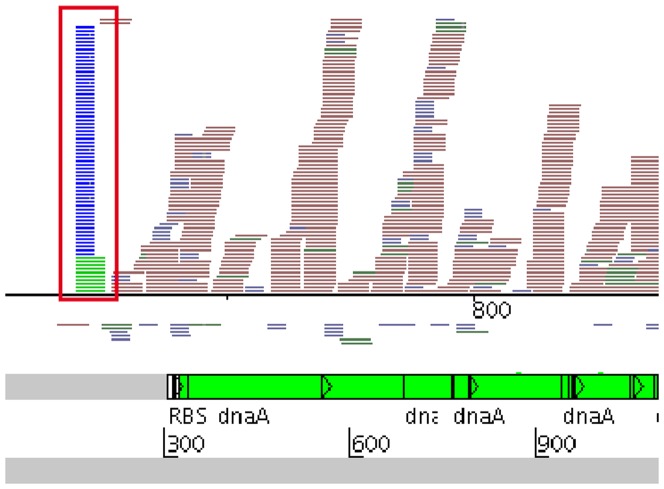
Pileup of reads representing the TSS of the *dnaA* gene of *L. monocytogenes*. Reads are mapped onto the *L. monocytogenes* genome and depicted as horizontal lines in the top half of the figure. Forward reads are mapped above, reverse reads below the base line. Blue reads are from the sample containing RNA fragments <40 nt, green reads from the sample containing RNA between 40 nt and 150 nt, red reads from the fraction containing RNA >150 nt. The lower half of the figure shows the corresponding annotation at this genome location, with the beginning of the *dnaA* gene at position 318. Artemis [39] was used to illustrate the mapped reads and annotation of the genome.

A specific pattern, creating a large pileup of reads with identical starting positions, located shortly upstream of annotated genes and operons, was a common structure seen in our data. Fig. 2 indicates such a read pattern before the gene *dnaA*. Its location and well-defined start was a hint, that these read patterns represent the transcription start sites (TSS) of the corresponding downstream gene or operon. An alignment of 20 randomly chosen samples of putative TSS from our data with TSS data from Wurtzel and colleagues [4] was performed to verify this assumption. Unfortunately it is impossible to clearly identify TSS solely based on the data at hand. However, we consistently found our putative TSS to be within 1 nt from those described by Wurtzel and coworkers [4], confirming that these patterns indicate TSS. Furthermore, we cannot distinguish between independent sRNAs and processed TSS's. Hence we removed all sRNAs identified as possible TSS from our later analysis.

### Identification and validation of sRNAs in the sequence data

**Figure 3:**
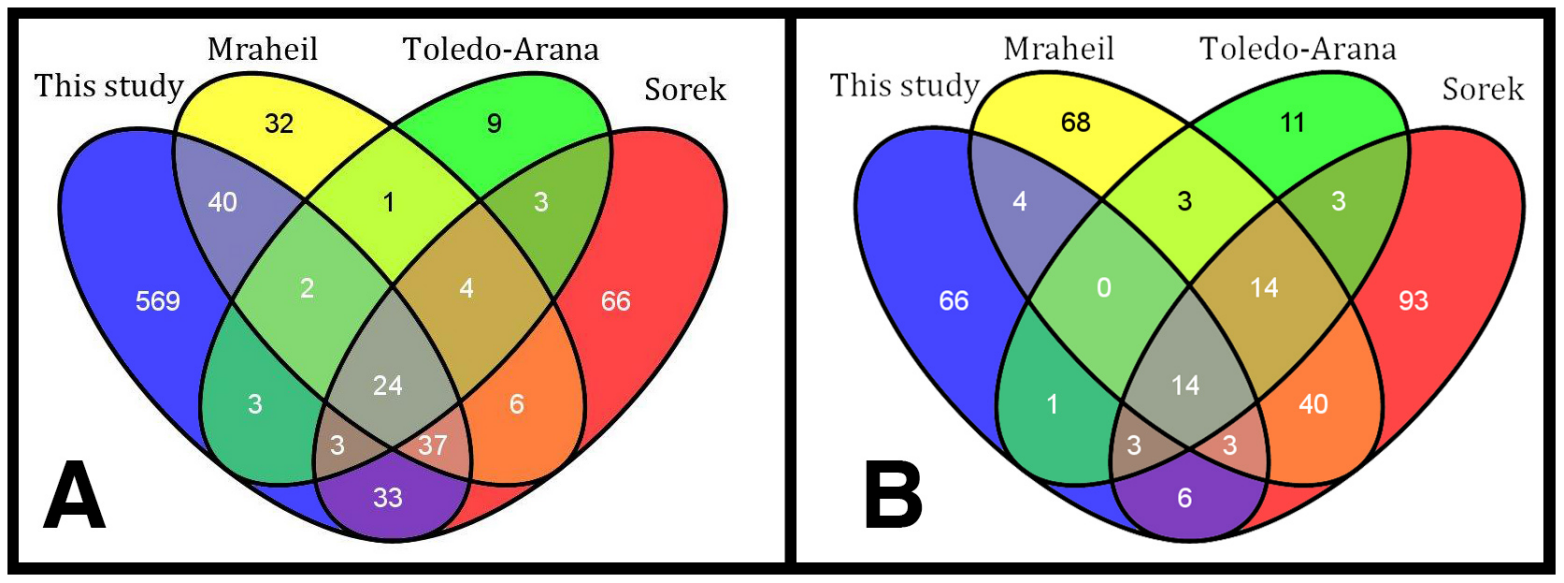
sRNAs identified by different studies [2]–[4] and this study and their overlap. sRNAs for this study were identified via automatic identification with our newly developed pipeline. 144 (55%) known sRNAs were recovered with the automated method. Of the 711 sRNAs identified in total, 569 were yet undescribed. The majority of these, however, were later removed due to their likely origin as transcription start site and 5′ UTR of known genes. Most of sRNAs, which were not recalled by the automated method, were found by manual reevaluation, increasing the total recall rate to 90%.

The high coverage with a total of 21 million SOLiD reads of 50 nt length enabled us to compare all of the 263 known sRNAs in *L. monocytogenes*, that were identified previously [2]–[4], [18], [20]. 142 of the 711 automatically identified sRNA candidates from this study were previously identified by three studies [2]–[4], as represented in Fig. 3. While these 142 (55%) known sRNAs were recovered by the automatic pipeline, a manual revision of known sRNAs specifically aiming at sRNAs, which were missed due to either the conservative coverage threshold applied or a filter discarding candidates too close to, or overlapping with annotated genes, increased the recovery rate to 90% of the previously described small RNAs in at least one of the two conditions and at least one of the 3 corresponding size fractions. When classifying the sRNAs automatically and manually according to their location and read patterns, we found 82 of the known sRNAs to represent UTRs of downstream genes rather than independently transcribed sRNAs in intergenic space. Furthermore, allowing for minor differences in size we found that most known sRNA match our findings. Notably, with all the differences between studies, there seemed to be a general consensus on the 5′ end of sRNAs, hence the transcription start site, often varying only by 1 or 2 nt, while the 3′ end and hence the transcription termination site of the same sRNA identified by different studies often varied extensively. Both, methodical limitation in the 3′ accuracy as well as biological variation due to unspecific termination of transcription may be a possible explanation for this observation. We summarized our findings in supplementary table S1, which contains a comprehensive list of known sRNAs and their features as well as their class indicated by our study.

The automated classification of sRNA candidates by our pipeline revealed that 70% of our sRNA candidates resemble TSS and long UTRs (>150 nt) instead of independent small transcripts. We removed those candidates and all known sRNAs from further analysis. The remaining 172 yet undescribed candidates where manually analyzed for their potential to resemble new sRNAs on the *L. monocytogenes* genome. Supplementary table S2 lists these 172 candidates and their individual automated and manual classification. Most of the 172 candidates identified by automated methods were dismissed after a manual inspection for one of several reasons: (1) probable origin as TSS, alternative TSS or 3′ UTR of a regular gene or annotated ORF, due to their location and read pattern, (2) expression below the local noise level, and (3) expression peaks on lowly expressed genes. The individual reasons to dismiss certain RNAs are also given in supplementary table S2. However, we propose nine new sRNAs candidates within the *L. monocytogenes* genome. These candidates show sufficient expression above the noise level and indications of independent expression.

### Nine new asRNAs

**Figure 4:**
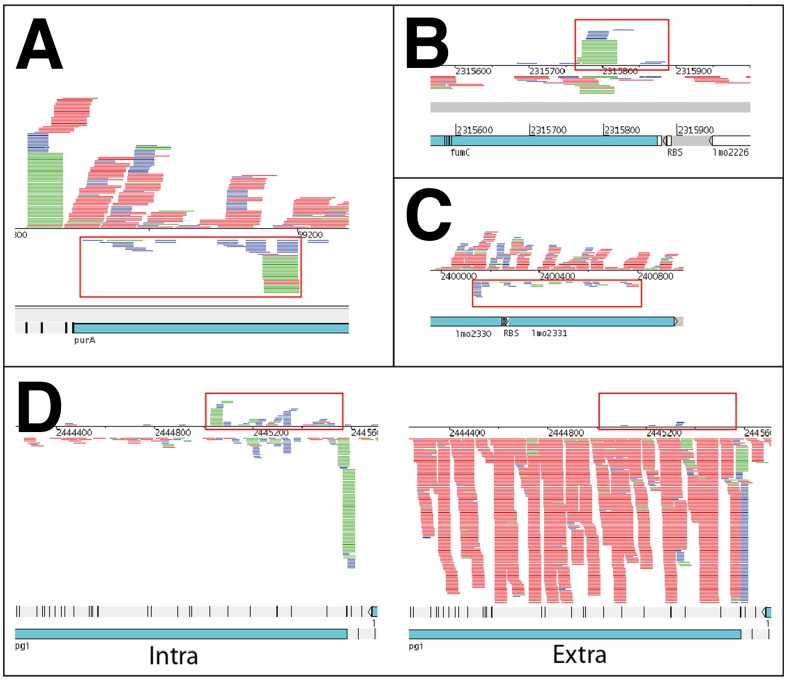
Pileup of reads representing four newly identified asRNAs of *L. monocytogenes*. Putative sRNAs are marked with red boxes. Each colored line represents a mapped read either on the forward strand (above the line) or the reverse strand (below the line). Blue reads are from the sample containing RNA fragments <40 nt, green reads from the sample containing RNA between 40 and150 nt. Red reads from the sample of RNAs >150 nt. The lower half of each figure shows the corresponding annotation at this genome location. (A) anti0055 (*purA*). Shown is the extracellular condition. (B) anti2225 (*fumC*). Shown is the extracellular condition. (C) anti2330 (*lmo2331*) in phage locus of *L. monocytogenes*. Shown is the extracellular condition. (D) anti2367 (*pgi*). Shown is the intracellular and extracellular condition respectively. Expression of the *pgi* gene and the boxed antisense RNA is mutual exclusive between the two conditions.

Analysis of the SOLiD sequencing data lead to the discovery of new small RNAs mostly transcribed anti-sense of annotated *L. monocytogenes* genes. We have picked nine candidates for further analysis. All nine candidates showed expression opposite of an annotated gene and therefore were classified as antisense RNAs. Fig. 4 and Fig. S1 show the read mappings of these nine asRNAs, which are listed in table 1. For some of the corresponding genes, a biological function is annotated, allowing us to infer a possible function of asRNAs.

**Table 1:** List of the nine newly identified sRNAs in *L. monocytogenes*, which are classified as asRNAs with the corresponding antisense gene given.

name	start	end	strand	length	class	corresponding gene
anti0055	59153	59203	−	50	asRNA	*lmo0055/purA*
anti0466	503060	503108	−	48	asRNA	*lmo0466*
anti2106	2186912	2187025	+	113	asRNA	*lmo2106*
anti2130	2213928	2213976	+	48	asRNA	*lmo2130*
anti2224-2	2314018	2314047	+	29	asRNA	*lmo2224*
anti2225	2315763	2315820	+	57	asRNA	*lmo2225/fumC*
anti2330	2400131	2400197	−	66	asRNA	*lmo2331*
anti2367	2445029	2445120	+	91	asRNA	*lmo2367/pgi*
anti2378	2454760	2454790	−	30	asRNA	*lmo2378*

Conservation analysis was performed using the MAUVE multiple genome alignment tool [25]. Of the nine candidates, most were well conserved within other *Listeria* species. anti0055 however, was specific for *L. monocytogenes* and anti2330 was found to be only conserved in *L. innocua and L. welshimeri*.

The asRNA anti0055 is located antisense of *lmo0055* or *purA*, an adenylosuccinate synthetase, important in the *de novo* synthesis of purine nucleobases, which also plays roles in infection [26] and intracellular growth [27]. Transcription of the antisense RNA starts 365 nt downstream of the TSS of *purA* in the opposite direction. The exact length of the transcript cannot be assessed, but additional reads downstream of the sRNAs TSS suggest a length of at least 289 nt. See Fig. 4(A) for read mappings in this locus. Significant expression of both, the *purA* gene as well as its asRNA can only be detected in the extracellular sample. Expression in the intracellular sample is very low and not above the expected noise level.

Another newly identified asRNA is transcribed opposite of *lmo2225*, a putative fumarate hydratase according to the KEGG database and based on orthology assumed to be active within the citrate cycle. Its putative TSS is 110 nt upstream of the beginning of the *fumC* gene, for which no independent TSS could be identified. Again, the length of the transcript cannot be determined with certainty, but additional reads suggest around 110 nt of length. Expression of anti_fumC can be found in intra- and extracellular sample. However expression is roughly 10-fold higher in the intracellular sample (see also Fig. 4(B)). Differential expression analysis found this locus to be differentially expressed with a p-value of 0.064. *L. monocytogenes* harbors a prophage locus with genes from *lmo2271* until *lmo2332* [28], which at the very end contains weak, but consistent expression of an antisense RNA. It covers parts of the genes *lmo2330* and *lmo2331* and stretches from near the 3′ end of *lmo2331* until the 3′ end of *lmo2330*. Expression can be detected in both extracellular and intracellular condition. See Fig. 4(C) for a mapping of reads onto the corresponding locus.

Most notably among the nine new asRNAs is anti2367 opposite of *lmo2367* or *pgi*, coding for a glucose-6-phosphate-isomerase with suggested function in the pentose-phosphate-pathway and glycolysis (see KEGG-database). Expression starts 568 nt upstream and on the opposite strand of the putative TSS for *pgi*. Its length can be estimated between 325 and 700 nt and expression can only be detected in the intracellular sample. Its differential expression p-value is 0.026 with a normalized fold change of 10.

### Experimental confirmation of novel asRNA candidates

**Figure 5:**
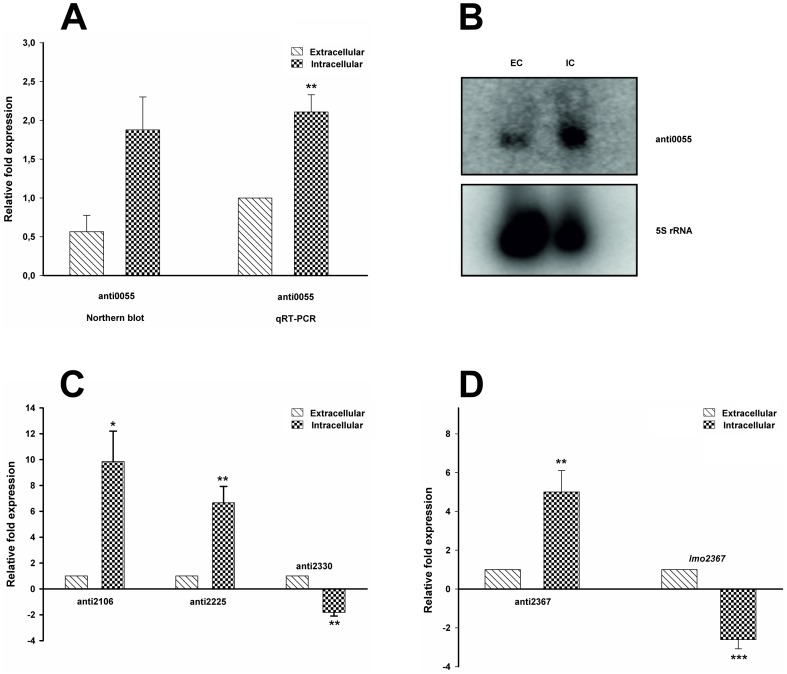
Validation of new asRNA transcripts from *L.* monocytogenes and their effect on gene regulation after transition to the intracellular growth conditions. A) The antisense RNA transcript anti0055 (*purA*) is validated by northern blot analysis and strand-specific qRT-PCR. The graph shows intracellular up-regulation of anti0055. B) Northern blot images of anti0055 and control 5S rRNA EC: Extracellular, IC: Intracellular. C) The presence of antisense transcripts anti2106 (*lmo2106*), anti2225 (*fumC*), and anti2330 (*lmo2330*) was determined by strand-specific qRT-PCR. anti2330 is down-regulated, anti2106 and anti2225 are up-regulated significantly. D) Strand-specific qRT-PCR analysis confirmed the existence and up-regulation of antisense RNA transcript anti2367. *pgi* (*lmo236*7) was down-regulated, which indicates the possible role of anti2367 in *pgi* gene regulation. ‘*’ P≤0.05; ‘**’ P≤0.01; ‘***’ P≤0.001.

To confirm the transcriptional regulation of several new asRNAs (≥50 nt) in our study we selected anti0055, anti2106, anti2225, anti2330 and anti2367 for performing qRT-PCR analysis. The results showed that all selected asRNAs are differentially expressed under intra- and extracellular growth conditions (see Fig. 5). In addition we could confirm by using northern blot analysis that anti0055 is up-regulated during intracellular growth (see Fig. 5(B)).

In the case of anti2673 which is up-regulated during intracellular growth, the corresponding gene *lmo2673* (*pgi)* on the other hand is down-regulated in the intracellular growth condition. See Fig. 4 (D) for the alignment of intracellular and extracellular reads to the *L. monocytogenes* genome, showing mutual exclusive expression of *pgi* and the corresponding asRNA.

### Long antisense RNAs

We were able to confirm the expression of five from six proposed lasRNAs in our sequence data and were able to identify asRNA candidates that have similar properties. These asRNAs have been previously reported, but in this study we found these are likely to resemble much longer lasRNAs. Specifically the asRNAs anti2046, anti2259, anti2677 and anti2717 all stretch over several genes and potentially form lasRNAs. Also see the comments of the corresponding asRNAs in supplementary table S1 for additional information on these lasRNAs. Supplementary Fig. S2 shows the mapping for all of the aforementioned possible lasRNAs in the artemis viewer.

## Discussion

Small RNAs in *L. monocytogenes* have been subject to intensive research over the last years. Improving technologies with increased sensitivity lead to the identification of 257 sRNAs in total by several studies using different techniques [2]–[4], [18], [20]. This study re-evaluates these small RNAs with focus on their probable origin and functional properties, and proposes nine new non-coding sRNAs, making use of an extensive transcriptome dataset, compiling a total of 21 million SOLiD sequencing reads. Five of these nine new asRNA could be confirmed via qRT-PCR and one candidate (anti0055) could also be validated in northern blot experiments by performing three biological independent experiments to show their biological relevance.

### Computational prediction of sRNAs by a new pipeline

We implemented a specialized analysis pipeline for the identification of sRNAs in SOLiD sequencing data. In contrast to existing pipelines and analysis tools, this pipeline exploits the specific properties of fractionated RNAseq data to identify sRNAs with increased sensitivity and specificity. The pipeline makes use of fractionated RNA data, to improve on the distinction between degradation products of large RNA molecules and independent small non-coding RNAs. Since distinction between long UTRs and sRNAs located 5′ of genes or polycistronic transcription and intergenic sRNAs is often inaccurate based solely on annotational data and read-pileup-shapes, a manual analysis of the data is still advised where the complete context of gene expression in an area can be assessed.

The pipeline was designed for use with SOLiD specific color-coded sequencing data as an input, but is easily usable with other next generation sequencing technologies as well, making it universally applicable. While it is possible to analyze and identify sRNAs based on a single RNA-Seq experiment with this pipeline, particularly projects with a multitude of datasets with RNA of different size fractions will strongly benefit from the pipelines capabilities of integrating information from between different datasets. Furthermore downstream analysis tools integrated into the pipeline help in the fast interpretation of acquired data. They include a clustering algorithm to identify the same sRNAs in different samples or studies, an automated sRNA classification system based on size, position, and read pattern of a candidate, as well as differential expression analysis to compare data taken under different conditions. The pipeline can be easily modified to meet a wide range of requirement for the analysis of transcriptomic data.

### lasRNA

Long antisense RNAs are a type of non-coding RNAs that have been described previously [3], [4]. These lasRNAs are significantly longer than typical, short asRNAs and typically stretch over whole genes instead of just covering the UTR of a gene. Wurtzel and colleagues proposed some of these lasRNAs have a double function both as mRNA and asRNA and introduced a related structure called excludon [4]. In this structure, two adjacent, yet oppositely arranged genes overlap with the other gene with their corresponding transcript and forms corresponding lasRNAs. This structure has the potential to create an expression regulation by mutual exclusion, where one gene cannot be expressed while the other is, as the transcript for one gene will also act as asRNA for the other.

We were able to identify four previously known asRNAs [3], [4] showing similar properties: anti2046, anti2259, anti2678 and anti2717 were all found to be significantly longer than originally proposed. All four candidates have been originally described to cover part of a single gene, but in our data were found to cover four to six genes instead. See corresponding comments in supplementary table S1 and the read pileups in supplemental Fig. S2. Given the length of the lasRNAs, structures comparable to the excludons described by Wurtzel and colleagues [4] are likely for these lasRNAs. The most likely reason for us to identify those sRNAs as significantly longer than before described, is the higher sequencing coverage in our experiments. It enables us to identify weekly but consistently transcribed areas better than before, leading to the discovery of previously unidentified long transcripts that were originally thought to be distinct or shorter.

### Identification of nine new sRNA candidates

Automated identification of asRNA in the data and manual refinement of results revealed nine new sRNAs candidates in *L. monocytogenes*. Most notably among these are four asRNAs opposite of the genes *lmo2225* (*fumC*), *lmo2330*, *lmo0055* (*purA)* and *lmo2367* (*pgi*).

The prophage A118 can be found in the *L. monocytogenes* EGD-e genome inserted between the genes *lmo2271* and *lmo2332* [22]. At the very end of this prophage region, covering the 3′ end of *lmo2331* and the 5′ end of *lmo2330* we identified another down-regulated asRNA (see Fig. 5 (C)). *lmo2331* is predicted to encode a cell wall lipoprotein, while *lmo2330* is similar to the phage protein *gp33*. Antisense transcription of the prophage genes has previously been reported and this might be an additional case of such [2]–[4]. Apart from this general antisense transcription it might represent specific and active repression of phage gene expression, as phage control by means of antisense transcription is a long known phenomenon [29]. More recently Irnov and colleagues also reported the expression of asRNA in prophages of *Bacillus subtilis* and suggested a function in maintaining the phage host equilibrium [14].

Antisense of the *purA* gene we were able to identify an asRNA at the 5′ end of the gene. The *purA* gene encodes a putative adenylosuccinate synthetase with assumed function in the de novo purine synthesis pathway, making it an essential enzyme in the synthesis pathway of purine nucleobases. Purine synthesis seemingly plays an important role for intracellular growth of *L. monocytogenes* [27] and a *L. monocytogenes* serotype 4b strain with a mutation of *purA* is known to be strongly attenuated in the infection of mice [26]. This makes a lifestyle dependent regulation of *purA* very likely, and asRNAs are known to play a major role in the adaption to rapid environmental changes in general [30] as well as the transition of *L. monocytogenes* from saprophytic to virulent lifestyle in particular [31]. However, no classical or obvious regulation pattern could be found when analyzing expression of both the *purA* gene and its corresponding asRNA within the RNA-Seq data which could be also observed by qRT-PCR (data not shown). We observed increased expression of asRNA anti0055 under intracellular versus extracellular growth condition using qRT-PCR analysis as well as northern blot analysis (see Fig. 5 (A and B)). The biological relevance of this up-regulated asRNA has to be characterized in future.

We identified a new asRNA anti2225 opposite of the *fumC* gene, coding for a fumarate hydratase typically with central function in the TCA-cycle. Interestingly, an antisense transcript of the homologous gene has also been found in the Gram-negative *Helicobacter pylori* and experimentally verified by northern blot and RT-PCR [32]. In addition, many asRNAs of housekeeping genes of *Cyanobacterium synechocystis* have been identified [33], demonstrating that such asRNAs are a common mechanism of transcriptional regulation. Furthermore *L. monocytogenes* is already suspected to have an interrupted TCA-cycle [34]. Also it shown that even an interrupted TCA-cycle may serve as an essential generator for purine for which we already propose a regulation by means of PurA [35]. Furthermore Schauer and coworkers have shown the central role of purine biosynthesis for intracellular growth [27]. Here we could show that expression of the *fumC* gene (data not shown) as wells as anti2225 (see Fig. 5(C)) is up-regulated after transition to the intracellular lifestyle. Biological interpretation of these finding is challenging at this point and needs further experimental validation. Signs of classical asRNA regulation patterns can be found expressed opposite of the gene *lmo2367/pgi* for anti2367. Inspecting the sequencing data of the intracellular and extracellular growth condition, the expression of either the gene or the asRNA seems to be mutually exclusive, giving a hint for a causal link and a possible regulation mechanism interfering with the expression of *pgi* on the transcriptional level. This pattern is clearly visible in Fig. 4(D) and Fig. 5(D) showing the mapped reads for both the intracellular and the extracellular condition which could be also confirmed by qRT-PCR analysis. Expression of the *pgi* gene is low for the intracellular growth, and high for the extracellular growth, while expression of the corresponding asRNA on the opposite strand is high for the intracellular and low for the extracellular condition (see Fig. 5(D)). *lmo2367*/*pgi*, encodes a glucose-6-phosphate isomerase with central function in the interface between glycolysis and the pentose phosphate pathway. Previous reports link the transition from extracellular to intracellular growth of *L. monocytogenes* to a reduced expression of *pgi* [36] and a corresponding shift in metabolic pathways leading to the degradation of glucose phosphate by the pentose phosphate pathway [1]. Furthermore a proteomic study was able to identify the *pgi* corresponding peptides under two different extracellular conditions but not within intracellular conditions of *L. monocytogenes* [37]. As a housekeeping gene, *pgi* is under the control of a housekeeping promoter, and hence requires promoter independent specific regulation of this gene. The identification of anti2367 sheds lights on the metabolic adaptation on transcriptional level by antisense RNAs in *L. monocytogenes*.

### Conclusion

The high coverage and strong strand specificity of our data revealed a substantial amount of general antisense transcription over the *L. monocytogenes* genome. Similar general antisense transcription has been described previously [33], [38]. The biological relevance of this phenomenon is not yet fully understood, but the finding of such in another bacterial organism underlines its importance of further inquiry of the matter. Given the high number of newly identified asRNAs as well as the identification of exceptionally long non coding antisense RNAs, lasRNAs, it is obvious that antisense transcription is an important factor in the regulatory network of *L. monocytogenes* and it should be investigated whether similar types of regulation are common in other bacterial species.

## Supporting Information

Table S1 **List of previously identified sRNAs.**
(XLS)

Table S2 **List of newly identified sRNAs.**
(XLS)

Table S3 **Oligonucleotides used in this study.**
(DOCX)

Table S4 **Reads count summary of experimental transcriptome data.**
(XLS)

File S1 **Detailed description of data analysis pipeline.**
(DOC)

Figure S1 **Read mapping of asRNA anti0466, anti2106, anti2130, anti2224-2 and anti2378.**
(PDF)

Figure S2 **Read mappings of lasRNA like structures.**
(PDF)
