# Supplementary material for: Ultra Deep Sequencing of Listeria monocytogenes sRNA Transcriptome Revealed New Antisense RNAs
Source: PLoS One. 2014 Feb 3;9(2):e83979. doi: 10.1371/journal.pone.0083979 (PMC3911899; doi:10.1371/journal.pone.0083979)
Supplement: Table S3 — Oligonucleotides used in this study. (DOCX) [file pone.0083979.s003.docx]

| **Table S3: Oligonucleotides used in this study.** | |  |
| --- | --- | --- |
|  |  |  |
| **Primer** | **Sequence 5' to 3'** | **Characteristics** |
| anti0055_nb | AATTTGCGTATTTCTAACCGTGCGC | Probe used for northern blot analysis |
| anti0055_a | TTCATGATAAAGGTGTGGATAC | Probes used for strand-specific qRT-PCR analysis of antisense RNAs |
| anti0055_b | GCGCACGGTTAGAAATACG |  |
| anti2106_a | AATGGGTGTGGTTAACTTA |  |
| anti2106_b | TAATAGGAATAGTCATAATCT |  |
| anti2225_a | AGCGCAAACAGAACGAAGTA |  |
| anti2225_b | GGCATAGGATTATCACCAATA |  |
| anti2330_a | TTGGGGGAGATAGCAATCATA |  |
| anti2330_b | CCAGCCATCTTTGTCTCGT |  |
| anti2367_a | TCCGTGTGTTCAAAGAACT |  |
| anti2367_b | AGCGCCTTTTGCTTTATCAG |  |
| lmo2367_a | GAATACTTGAGGTGTTTTACGC | Probes used for strand-specific qRT-PCR analysis of *pgi* gene |
| lmo2367_b | ACAGCGACTCCGATGTATTA |  |
