# Supplementary material for: Ultra Deep Sequencing of Listeria monocytogenes sRNA Transcriptome Revealed New Antisense RNAs
Source: PLoS One. 2014 Feb 3;9(2):e83979. doi: 10.1371/journal.pone.0083979 (PMC3911899; doi:10.1371/journal.pone.0083979)
Supplement: File S1 — Detailed description of data analysis pipeline. (DOC) [file pone.0083979.s005.doc]

**Supplementary file S3: Detailed description of data analysis pipeline**

**Mapping reads to reference genomes**

In order to reconstruct the expression profile of the transcriptome we mapped the short sequence reads against the reference genomes described earlier.

We used the Burrows-Wheeler Alignment Tool (BWA) [22] to map SOLiD reads to references. We followed the default method described in the manual found at

<http://bio-bwa.sourceforge.net/bwa.shtml>

The reference genome was indexed in a first step. Indexing is done to speed up the mapping process, when multiple datasets are mapped against a single reference.

BWA can handle color space encoded reads as generated by SOLiD, but needs them to be in pseudo nucleotide-coding for its input. This means colors are encoded by standard nucleotide letters {a,c,t,g} instead of the digits {1,2,3,4}. Thus, the input fastq files were converted to pseudonucleotide-coding prior to usage with BWA. This was done using a solid2fastq script included in the BWA package. Mapping was performed using the parameter -i/--mismatch SW Mismatch Score, set to a value of 2.

**Identifying possible small RNAs: Overview**

At this step of the pipeline, we mapped reads to their corresponding position on the L. monocytogenes genome. The next task is to identify expressed regions of interest, i.e. those regions harboring potentially regulatory sRNAs. In order to do so, we first remove all reads corresponding to long transcripts of known and annotated genes. Furthermore we expect a great amount of decay product of the very same large transcripts, which we also want to remove from the data set. The details of each step is described in the following paragraphs.

**Identification of continuously covered regions**

For the following procedures we need an algorithm identifying continuously covered regions in a mapping. This procedure is key to both, the mapping filter and the candidate identification method. We implemented a module in the ncFinder to identify such regions based on the following parameters:

• length of the regions

• peak coverage of a region

• number of reads in a region

• mean coverage of a region

• allowed gap size within a region

The implemented algorithm will use an extending window approach, where a regions size is extended as long as it has at least a coverage of one, and the maximum allowed gap is not exceeded. A coverage of one is used to identify the largest possible fragmet size. If a region cannot be exceeded anymore because there is no more coverage, it is tested whether it meets the other criteria given. These include mean coverage and length. The region will be reported as hit if it does meet all criteria, and dismissed otherwise.

**Filtering reads originating from long transcripts**

We wanted to exclude all reads originating from long transcripts of known genes and their degradation products from further analysis. We thus implemented two filters based on annotation of the L. monocytogenes genome and the information within our data. These filters were then used to create masked regions in the genome. Reads falling within a masked region will be ignored in all subsequent analysis steps. The first filter is aimed at removing all reads falling within a region of an annotated gene. The annotation dependent filter is described in the next paragraph. The second aims to identify expressed transcripts in the data set and and filter the corresponding degradation product in the smaller fractions. It is described in more detail in the paragraph “Expression dependent filtering“.

Reads were considered to lie within a masked region, if their 5’ end lied within that region. The rationale behind that is, that for reads corresponding to long transcript or its decay products, we expect that they all start within the region of the long transcript, but not necessarily end within this region. If one considers a long transcript and its corresponding reads, they all must start within the range of the transcript. reads starting downstream of a putative transcript cannot originate from this transcript. Furthermore, reads staring upstream of a putative transcript, but overlapping with the 5’ end of this long transcript also cannot be originating from this transcript. Thus all reads beginning within the range of a putative transcript, but not necessarily ending in such a region, are removed.

**Annotation dependent filtering**

The L. monocytogenes genome annotation used for this study was obtained on 28/09/2011 from the NCBI.

<ftp://ftp.ncbi.nih.gov/genomes/Bacteria/Listeria_monocytogenes_EGD_e_uid61583/>

We obtained all ’gene’ and ’CDS’ features from the gff file and categorized them according to length. Features smaller than 150nt were masked in the middle fraction used to create the first, features larger that 150nt were used to create the second filter. We added a 50nt 3’ and 5’ to each feature to account for 5’ and 3’ UTRs of transcripts. The first filter was then applied to the middle, the second to the large fractions. We did not create a separate filter for the small fraction, as there are no such short features found in the annotation.

**Expression dependent filtering**

The second method identifies large transcripts based on coverage and masks the respective regions for smaller-size fractions, as reads falling in that region are considered to represent degradation products from the larger transcript. In a first step, continuously covered regions are identified with the method described previously. Parameter values were set to allow minimum average coverage of 3, a minimum number of reads of 6 and a maximum gap of 3nt. Additionally regions had to be at least 150nt and 40nt in length for large and middle region respectively to be considered a single transcript. The filter created on the large fraction, was subsequently applied to the middle and the small fraction, and the filter created on the middle fraction was applied on the small fraction. After removal of all reads corresponding to long and known transcripts, we now want to extract regions of interest from the remaining reads. To do so we again identified continuously covered regions in the remaining mapping. Regions were identified for each dataset using a minimum number of reads of 10 and a minimum mean coverage of 2. The resulting regions represent the first list of possible small RNA candidates.

**Clustering regions**

In order to identify possible hits in the different data sets and earlier studies in an unambiguous way we developed an ID system based on a locus specific clustering of the candidates. The clustering algorithm calculated an all against all matrix to find overlaps between regions. Regions were considered to correspond to the same transcript, if at least 50% of one of the two regions overlapped with the other. If two regions were found to be equal, they were both added to a single cluster, thus a single candidate. In an iterative process, all subsequent regions similar to one of a clusters member are added to this cluster. We applied the clustering algorithm on a pooled dataset including regions from datasets of all fractions, conditions and mappers. Additionally we included the regions of previously published small RNAs by Mraheil et al. (2011) and Toledo-Arana et al. (2009) in the clustering. Subsequently we assigned IDs to each cluster and the contained regions. In case the cluster contained sRNAs by Mraheil or Toledo-Arana, their original id was inherited. In case of a cluster containing both, regions from Toledo and Mraheil, we inherited the id of the older study by Toldeo-Arana. All clusters containing no previously published sRNAs were given sequential ids.

**Classification of candidates**

We classified our candidates based on read patterns extracted by visual analysis of a sample subset of all candidates. For each pattern identified, we inferred rules for the automatic identification by a classifier. We then implemented the classifier based on these rules and applied it on the complete set of candidates. Here we will describe the exact rules to classify candidates inferred from visual analysis. Identification of sRNA classes From each dataset we randomly selected 5 candidates, creating a list of 30 candidates in total. Each candidate was examined over all three size fractions to infer specific read patterns and location relative to other transcripts or anno- tated genes. The classifier was implemented as part of the ncFinder package. The corresponding classes can be found in the package edu.at.univie.csb.behrens.ncFinder. regions.classification the classifier can be executed with the script classify in the pipeline package distributed with this work.

**Rules for the classification of candidates**

After visual inspection we compiled a list of 4 common classes of sRNAs identified by the following rules.

**5PRIME**

The 5’ class is characterized by its sharp 5’ edge and its location shortly 5’ of an annotated gene (hence the name). Small RNAs classified as 5’ had to present the following characteristics:

1. an increase in coverage of at least 5 within a single nucleotide

2. an annotated gene within 300nt downstream of the beginning of the candidate

3. there is no other region downstream of the region until the annoted gene, that fulfills these criteria

**3PRIME**

The 3’ class has similar read patterns as the 5’ class, however its location is downstream of a gene, not upstream. The exact rules for their classification were as follows:

1. an increase in coverage of at least 5 within a single nucleotide

2. an annotated gene within 100nt upstream of the beginning of the candidate

3. there no other region upstream of the region until the gene, that fulfills these criteria

**ANTISENSE**

Candidates of the antisense class are identified by their antisense orientation relative to annotated genes. As asRNAs are frequently transcribed antisense of the UTRs of genes, we allow some overlap of the corresponding annotated CDS. We applied the following rules to identify antisense sRNAs:

1. genes and CDS are expanded by 250nt upstream and 150nt downstream to account for the 5’ and 3’ UTR respectively

2. the region fully overlaps with an annotated gene or CDS

3. the regions orientation is inverse to the annotated gene or CDS

**SENSE**

Sense candidates are characterized by their location within a coding sequence or CDS and with the same orientation. To avoid dual classification as 5’ and sense or 3’ and sense, we required them to overlap entirely with the gene or CDS. Hence the following single rule identified sense sRNAs:

1. the region fully overlaps with an annotated gene or CDS in the same orientation

**Text extracted and slightly variated from:**

Sebastian Behrens. “Identification of small RNAs in Listeria monocytogenes”, Master Thesis, Technical University of Munich, 2012
