# Supplementary figures and images for: Ultra Deep Sequencing of Listeria monocytogenes sRNA Transcriptome Revealed New Antisense RNAs
Source: PLoS One. 2014 Feb 3;9(2):e83979. doi: 10.1371/journal.pone.0083979 (PMC3911899; doi:10.1371/journal.pone.0083979)

anti0466

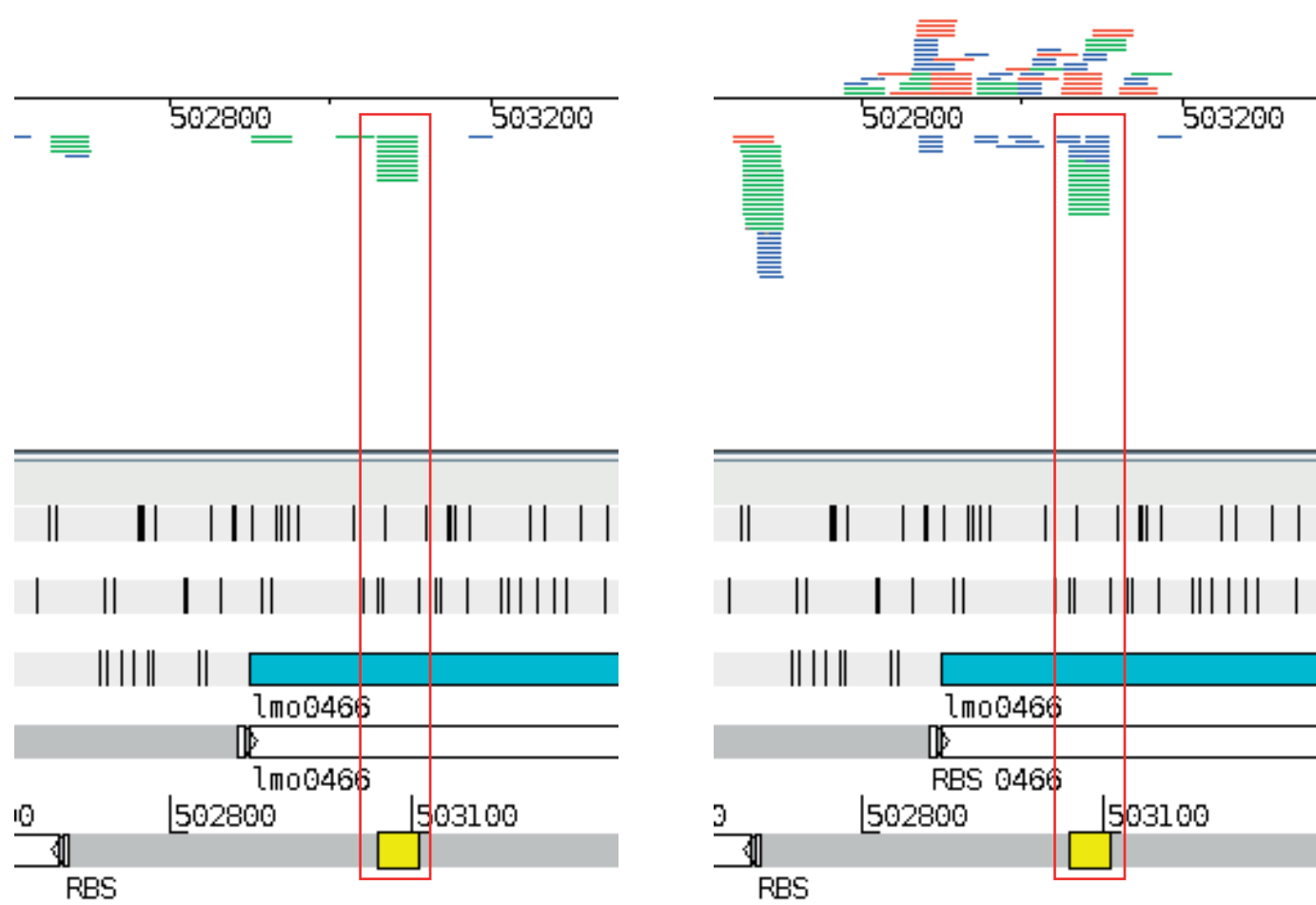

anti2106

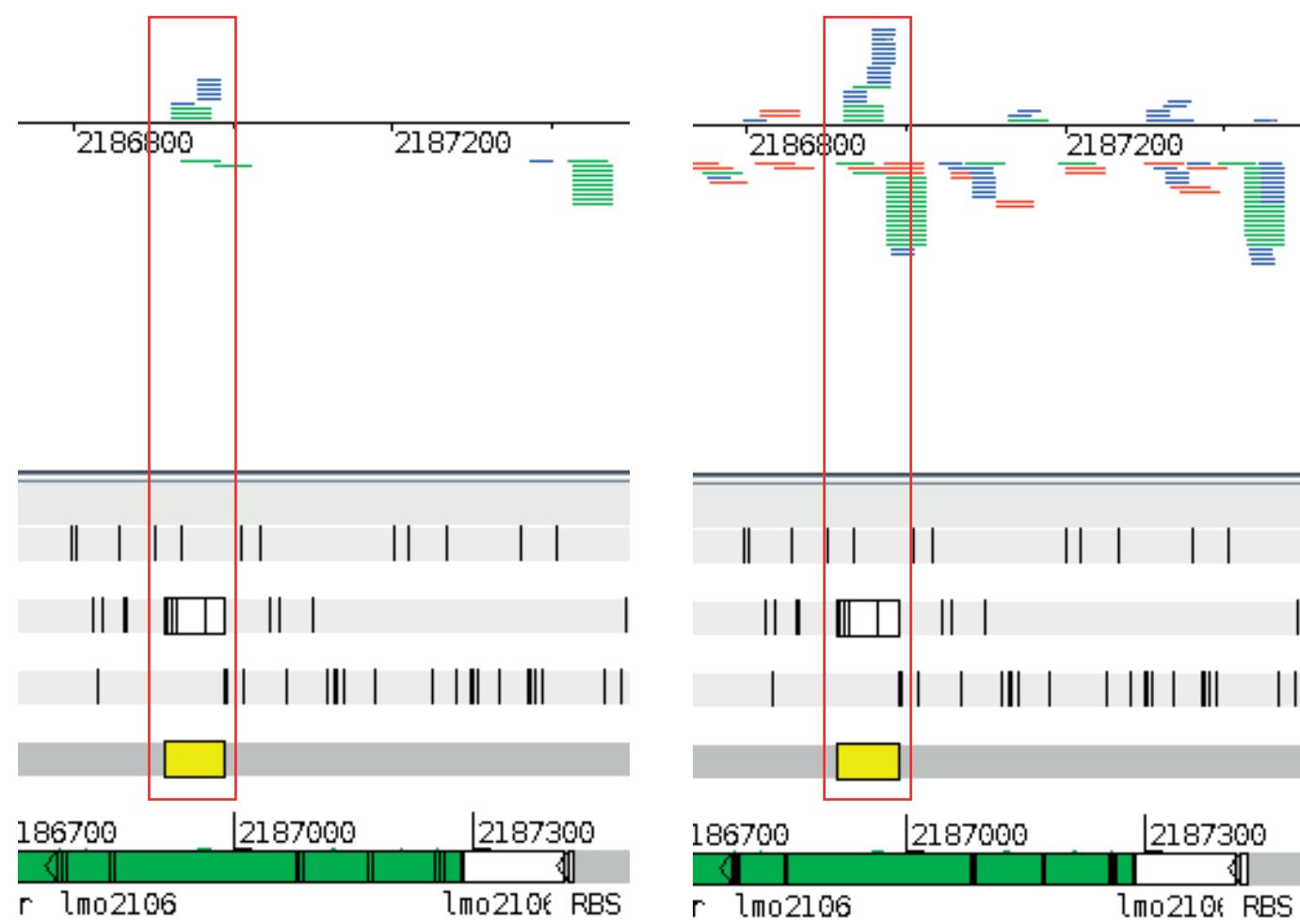

anti2130

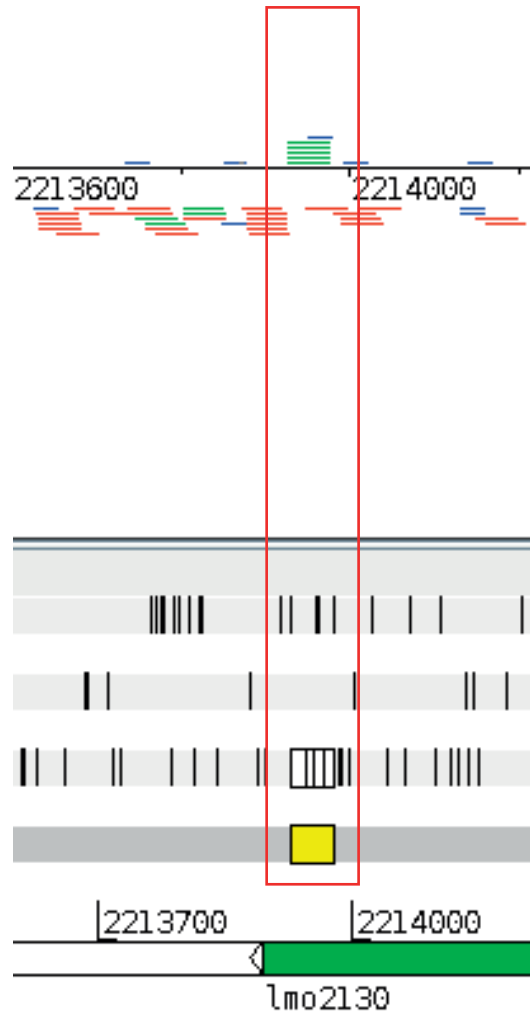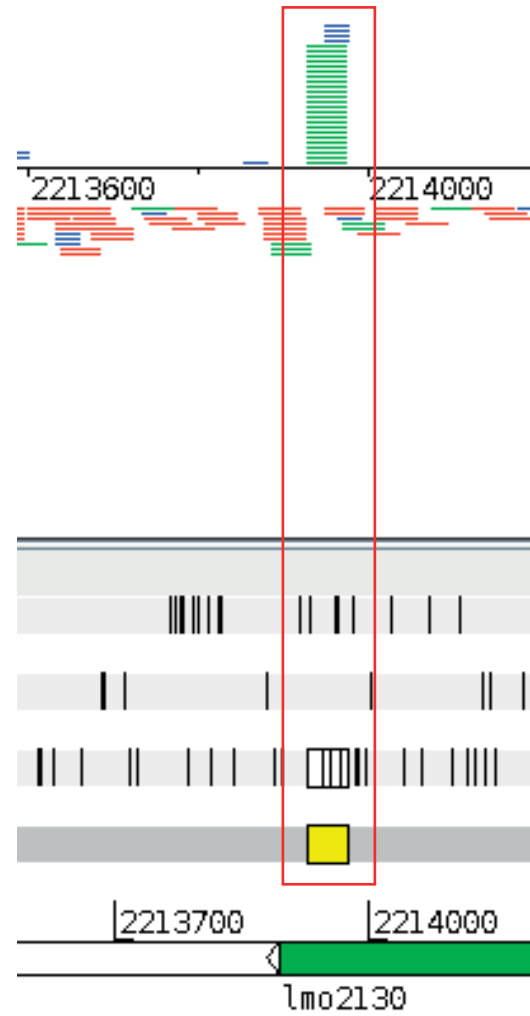

anti2224

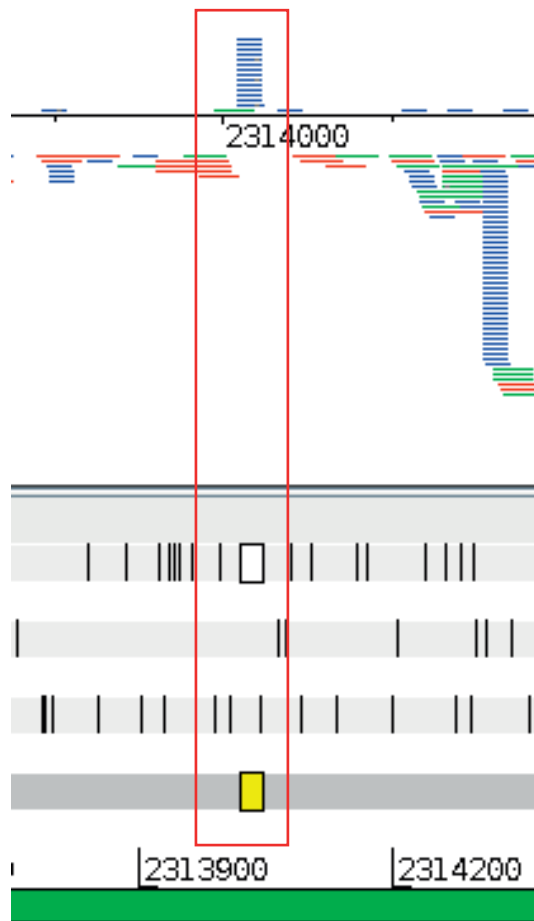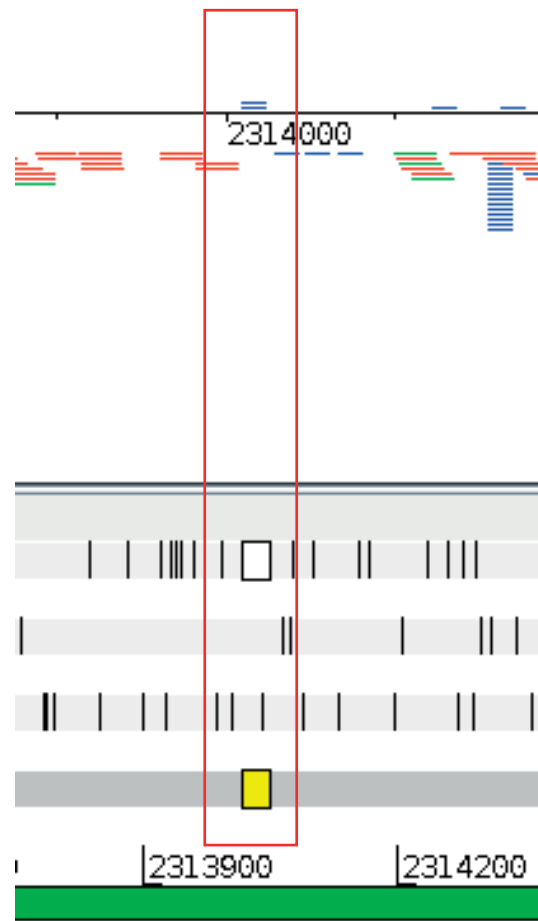

anti2378

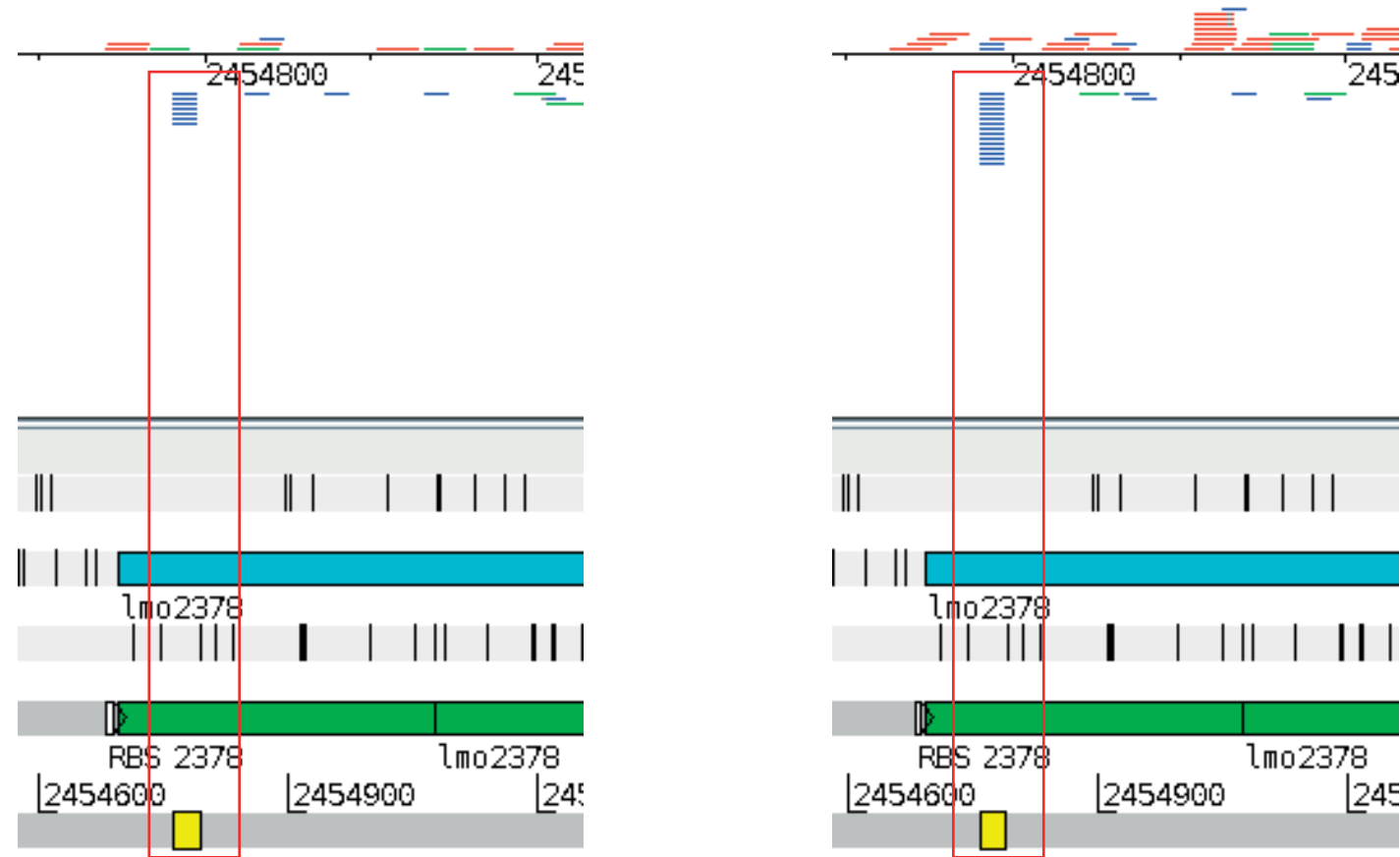

Supplement: Figure S1 — Read mapping of asRNA anti0466, anti2106, anti2130, anti2224-2 and anti2378. (PDF) [file pone.0083979.s006.pdf]
