# Supplementary material for: Ultra Deep Sequencing of Listeria monocytogenes sRNA Transcriptome Revealed New Antisense RNAs
Source: PLoS One. 2014 Feb 3;9(2):e83979. doi: 10.1371/journal.pone.0083979 (PMC3911899; doi:10.1371/journal.pone.0083979)

anti2046, extracellular

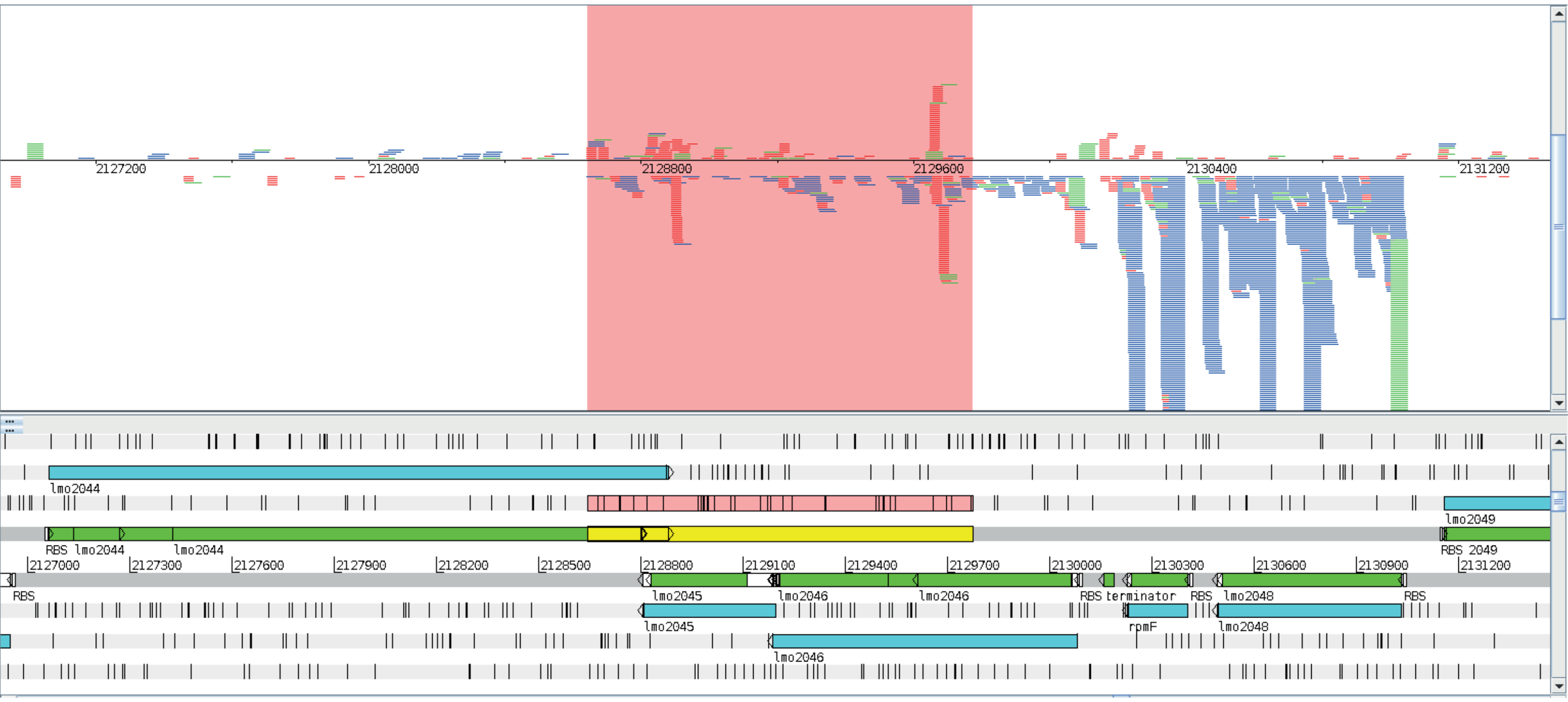

anti2259, extracellular

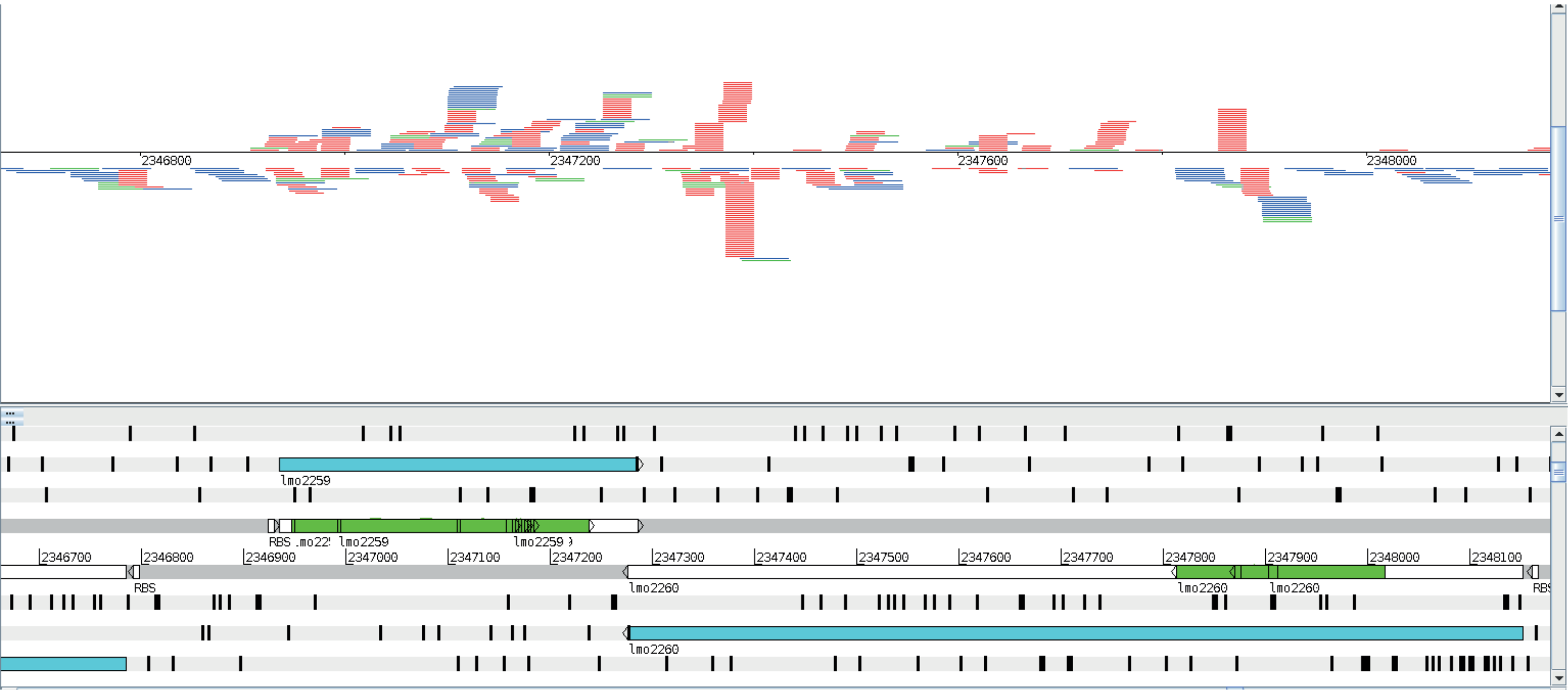

anti2677, extracellular

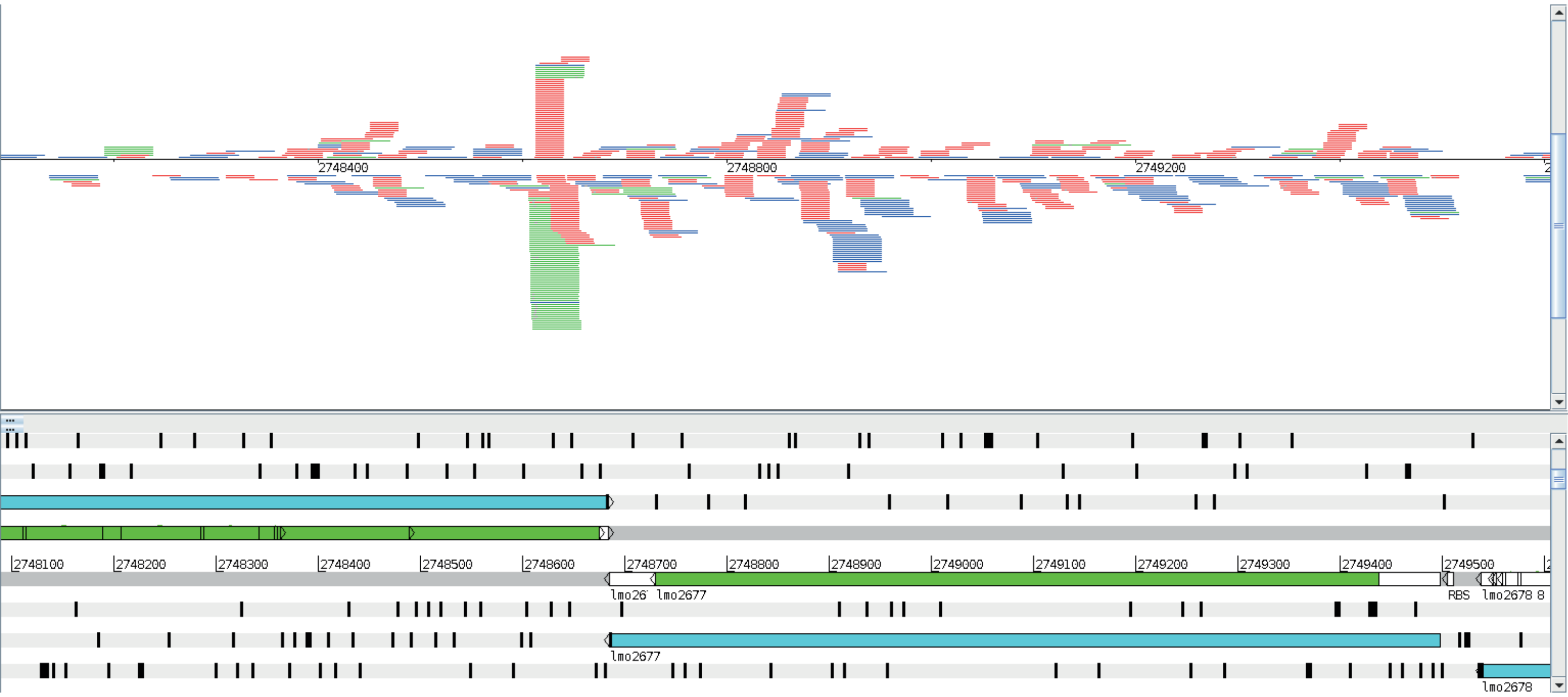

anti2677, intracellular

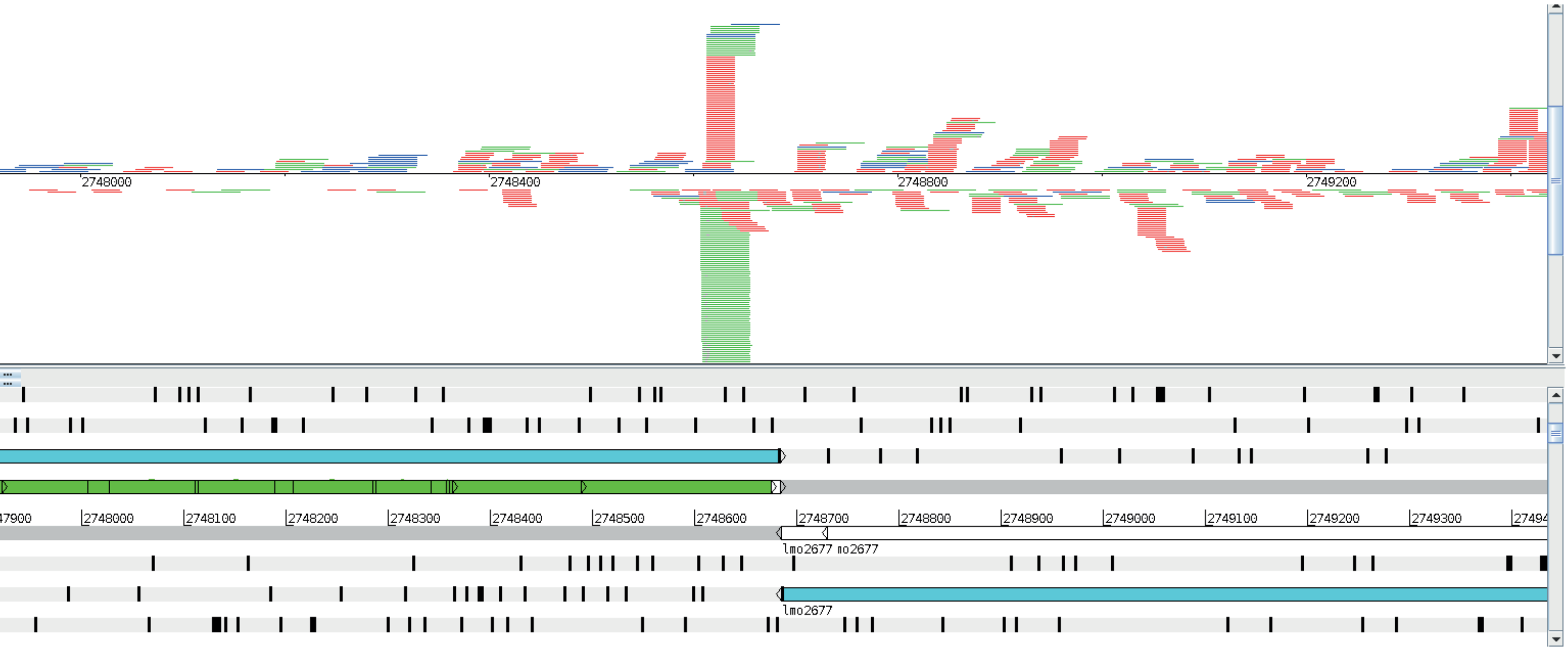

anti2717, extracellular

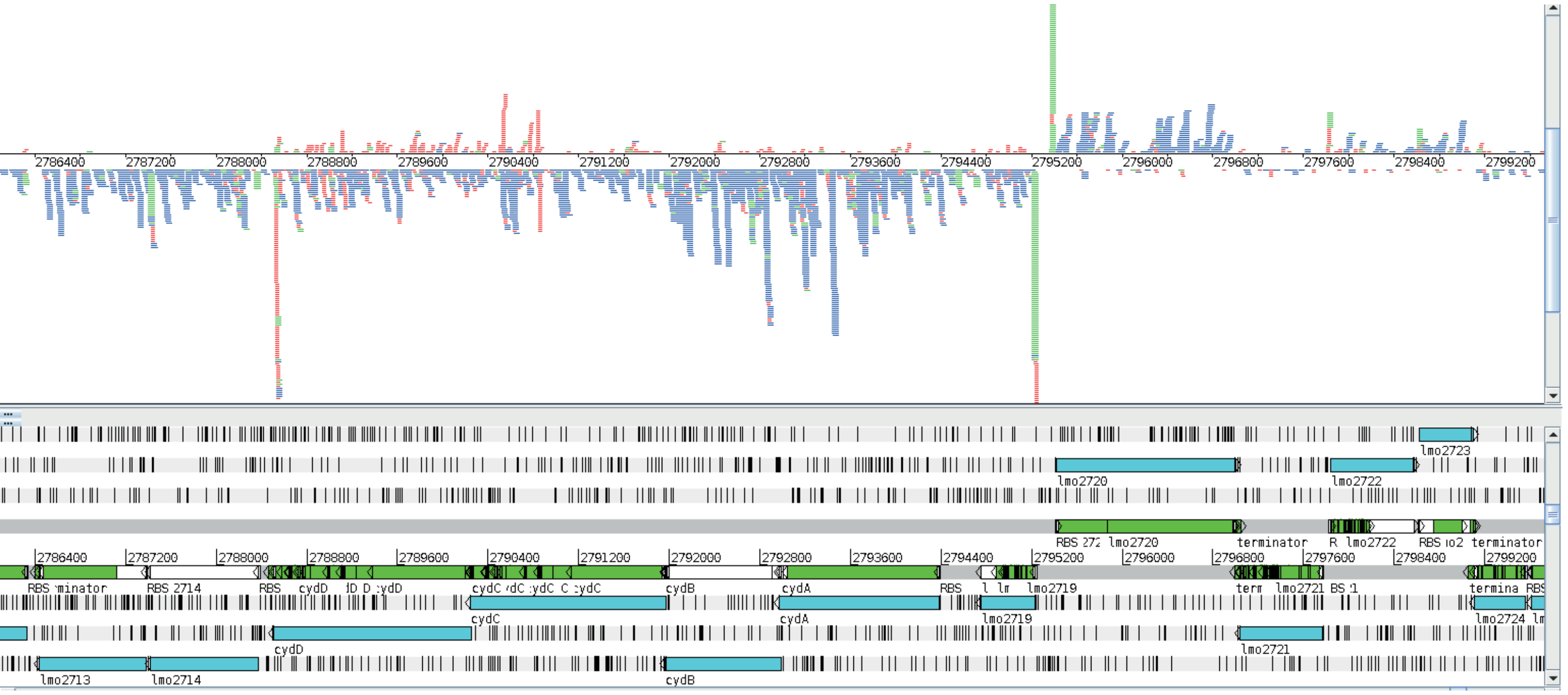

Supplement: Figure S2 — Read mappings of lasRNA like structures. (PDF) [file pone.0083979.s007.pdf]
